# Supplementary material for: Dietary Fiber, Whole Grains, and Head and Neck Cancer Prognosis: Findings from a Prospective Cohort Study
Source: Nutrients. 2019 Sep 27;11(10):2304. doi: 10.3390/nu11102304 (PMC6835374; doi:10.3390/nu11102304)
Supplement: Supplementary file 1 [file nutrients-11-02304-s001.pdf]

Online Supplemental Material

Table S1. Results of Multivariable Cox regression models for survival after mode imputation of HPV status. Individuals with a status denoted as unknown were assigned to the most prevalent category (HPV-negative).

**Fiber intake quintile and range (g/day)**	1<br><12.94	2<br>12.94-15.87	3<br>15.90-19.00	4<br>19.05-22.91	5<br>>22.91
**All-Cause Mortality**					
[1]Model 2	Referent	0.80 (0.46-1.37)	0.58 (0.33-1.03)	0.77 (0.45-1.33)	**0.42 (0.22-0.80)****
[2]Model 3	Referent	0.83 (0.43-1.59)	0.63 (0.31-1.25)	0.68 (0.30-1.51)	**0.37 (0.14-0.95)***
**Cancer-Specific Mortality**					
[1]Model 2	Referent	1.01 (0.51-2.01)	0.80 (0.39-1.64)	0.83 (0.41-1.69)	0.63 (0.28-1.41)
[2]Model 3	Referent	1.10 (0.48-2.51)	0.81 (0.34-1.96)	0.69 (0.24-1.94)	0.46 (0.14-1.53)
**Whole grains intake quintile and range (g/day)**	1<br><13.70	2<br>13.71-23.41	3<br>23.43-32.95	4<br>32.96-44.29	5<br>>44.29
**All-Cause Mortality**					
[1]Model 2	Referent	0.88 (0.53-1.47)	0.60 (0.33-1.10)	0.71 (0.40-1.25)	0.65 (0.37-1.15)
[2]Model 3	Referent	0.85 (0.50-1.46)	0.63 (0.33-1.20)	0.88 (0.47-1.67)	0.64 (0.34-1.23)
**Cancer-Specific Mortality**					
[1]Model 2	Referent	1.12 (0.57-2.21)	0.96 (0.45-2.04)	0.98 (0.48-2.02)	0.88 (0.41-1.88)
[2]Model 3	Referent	1.17 (0.57-2.40)	0.92 (0.41-2.08)	1.24 (0.55-2.77)	0.84 (0.36-1.96)

*** p <0.05**
**** p <0.01**

[1] Clinicopathological model—Multivariable Cox proportional hazards model fit with the following covariates: sex, age, HPV status, tumor stage, tumor site.
[2] Fully adjusted model—Multivariable Cox proportional hazards model fit with the following covariates: sex, age, HPV status, tumor stage, tumor site, education status, mean fruit and vegetable consumption, glycemic load, total fat, BMI, smoking, and drinking status.

Table S2. Results of Multivariable Cox regression models for recurrence after mode imputation of HPV status. Individuals with a status denoted as unknown were assigned to the most prevalent category (HPV-negative).

**Fiber intake quintile and range (g/day)**	1<br><12.94	2<br>12.94-15.87	3<br>15.90-19.00	4<br>19.05-22.91	5<br>>22.91
**Recurrence**					
[1]Model 2	Referent	1.31 (0.73-2.34)	0.97 (0.53-1.76)	0.85 (0.45-1.59)	0.93 (0.49-1.77)
[2]Model 3	Referent	1.42 (0.73-2.75)	0.98 (0.49-1.98)	0.74 (0.32-1.73)	0.77 (0.30-1.99)
**Whole grains intake quintile and range (g/day)**	1<br><13.70	2<br>13.71-23.41	3<br>23.43-32.95	4<br>32.96-44.29	5<br>>44.29
**Recurrence**					
[1]Model 2	Referent	0.96 (0.55-1.67)	0.72 (0.39-1.33)	0.86 (0.48-1.55)	0.80 (0.44-1.47)
[2]Model 3	Referent	1.06 (0.59-1.92)	0.77 (0.40-1.50)	1.06 (0.56-2.03)	0.76 (0.38-1.49)

[1] Clinicopathological model—Multivariable Cox proportional hazards model fit with the following covariates: sex, age, HPV status, tumor stage, tumor site.
[2] Fully adjusted model—Multivariable Cox proportional hazards model fit with the following covariates: sex, age, HPV status, tumor stage, tumor site, education status, mean fruit and vegetable consumption, glycemic load, total fat, BMI, smoking, and drinking status.

Table S3. Multivariable hazard ratios and their 95% confidence intervals of mortality risk by pretreatment total dietary fiber consumption quintiles, stratified by sex, stage, BMI, smoking status, and tumor site

**Fiber intake quintile**[1]	1	2	3	4	5	*P*-trend[2]
**Sex**						
Males (*n* = 344)	Referent	0.78 (0.37-1.66)	0.73 (0.34-1.58)	0.60 (0.23-1.54)	0.41 (0.13-1.27)	0.13
Females (*n* = 117)	Referent	0.15 (0.02-1.35)	0.05 (0.00-0.89)	0.28 (0.03-2.94)	0.21 (0.02-2.07)	0.39
**Stage**						
Stage 0, I,II (*n* = 165)	Referent	0.21 (0.02-2.07)	0.82 (0.06-12.11)	0.67 (0.04-11.36)	0.30 (0.01-6.54)	0.57
Stage III, IV (*n* = 318)	Referent	0.88 (0.43-1.78)	0.89 (0.43-1.85)	0.35 (0.14-0.85)	0.32 (0.11-0.97)	0.02
**BMI (kg/m^2)**						
BMI < 25) (*n* = 160)	Referent	0.61 (0.22-1.72)	0.31 (0.09-1.12)	0.24 (0.04-1.25)	0.16 (0.02-1.12)	0.07
BMI ≥25 (*n* = 303)	Referent	1.59 (0.63-3.98)	1.44 (0.55-3.79)	0.99 (0.34-2.91)	0.70 (0.20-2.44)	0.41
**Smoking Status**						
Current/former (*n* = 330)	Referent	0.73 (0.34-1.50)	0.68 (0.33-1.43)	0.50 (0.21-1.19)	0.50 (0.18-1.35)	0.006
Never (*n* = 132)	Referent	0.01 (0.00-0.69)	0.04 (0.00-5.97)	0.01 (0.00-0.21)	0.01 (0.00-0.21)	0.02
**Tumor Site**						
Oropharynx (*n* = 184)	Referent	0.54 (0.13-2.32)	0.67 (0.16-2.77)	1.39 (0.24-8.07)	0.24 (0.03-2.17)	0.36
Oral Cavity (*n* = 173)	Referent	2.41 (0.87-6.64)	0.78 (0.22-2.73)	0.40 (0.10-1.56)	0.22 (0.05-1.11)	0.02
Larynx (*n* = 94)	Referent	0.25 (0.03-2.30)	0.43 (0.07-2.80)	0.72 (0.05-9.60)	0.36 (0.24-9.06)	0.35

*** *p* <0.0045** [3]

[1] Stratified models adjusted for sex, age, HPV status, tumor stage, tumor site, glycemic load, total fat, mean fruit and vegetable consumption, education status, BMI, smoking, and drinking status.
[2] *p* value for a test of linear trend. Participant dietary fiber intake was set to the median of the subject's respective quintile. This variable was subsequently modeled as a continuous term using Cox regression.
[3] The level of $\alpha = 0.0045$ was adjusted using Holm-Bonferonni method for multiple comparisons.

Table S4. Multivariable hazard ratios and their 95% confidence intervals of mortality risk by pretreatment total whole grains consumption quintiles, stratified by sex, stage, BMI, smoking status, and tumor site.

**Whole grains intake quintile**[1]	1	2	3	4	5	*P*-trend[2]
**Sex**						
Males (*n* = 344)	Referent	0.78 (0.42-1.43)	0.59 (0.28-1.26)	0.75 (0.35-1.59)	0.67 (0.31-1.47)	0.40
Females (*n* = 117)	Referent	1.51 (0.22-10.39)	3.37 (0.33-33.76)	30.91 (3.92-245.18)	0.61 (0.07-5.63)	0.91
**Stage**						
Stage 0, I,II (*n* = 165)	Referent	1.06 (0.23-4.85)	0.82 (0.14-4.65)	1.68 (0.32-8.90)	0.31 (0.04-2.20)	0.42
Stage III, IV (*n* = 318)	Referent	0.77 (0.40-1.45)	0.80 (0.41-1.58)	0.97 (0.48-1.97)	0.43 (0.20-0.90)	0.06
**BMI (kg/m^2)**						
BMI < 25) (*n* = 160)	Referent	0.90 (0.35-2.32)	1.26 (0.48-3.30)	1.30 (0.41-4.14)	0.52 (0.15-1.87)	0.53
BMI ≥25 (*n* = 303)	Referent	1.03 (0.48-2.23)	0.60 (0.23-1.56)	1.53 (0.66-3.58)	0.62 (0.26-1.44)	0.40
**Smoking Status**						
Current/former (*n* = 330)	Referent	0.74 (0.39-1.41)	0.63 (0.32-1.25)	1.10 (0.56-2.16)	0.66 (0.32-1.35)	0.09
Never (*n* = 132)	Referent	0.42 (0.04-4.79)	0.04 (0.00-1.34)	0.01 (0.00-0.41)	0.11 (0.01-2.02)	0.36
**Tumor Site**						
Oropharynx (*n* = 184)	Referent	2.60 (0.69-9.85)	0.51 (0.11-2.40)	1.53 (0.33-7.00)	1.22 (0.27-5.65)	0.60
Oral Cavity (*n* = 173)	Referent	1.36 (0.52-3.58)	1.99 (0.63-6.27)	1.08 (0.38-3.07)	0.63 (0.21-1.91)	0.15
Larynx (*n* = 94)	Referent	0.50 (0.07-3.58)	0.31 (0.04-2.27)	0.76 (0.08-6.94)	1.71 (0.16-18.10)	0.42

*** p <0.0045** [3]

[1] Stratified models adjusted for sex, age, HPV status, tumor stage, tumor site, glycemic load, total fat, mean fruit and vegetable consumption, education status, BMI, smoking, and drinking status.
[2] p value for a test of linear trend. Participant whole grains intake level was set to the median of the subject's respective quintile. This variable was subsequently modeled as a continuous term using Cox regression.
[3] The level of $\alpha = 0.0045$ was adjusted using Holm-Bonferonni method for multiple comparisons.

Table S5. Multivariable hazard ratios and their 95% confidence intervals of recurrence risk by pretreatment total dietary fiber consumption quintiles, stratified by sex, stage, BMI, smoking status, and tumor site.

**Fiber intake quintile**[1]	1	2	3	4	5	*P*-trend[2]
**Sex**						
Males<br>(*n* = 344)	Referent	1.33 (0.61-2.86)	1.06 (0.48-2.36)	0.50 (0.18-1.41)	0.61 (0.19-1.93)	0.25
Females<br>(*n* = 117)	Referent	1.75 (0.12-4.61)	0.37 (0.04-3.32)	1.79 (0.188-17.06)	0.87 (0.11-6.92)	0.63
**Stage**						
Stage 0, I,II<br>(*n* = 165)	Referent	1.89 (0.29-12.08)	2.72 (0.23-32.60)	2.70 (0.22-33.54)	2.26 (0.18-27.98)	0.72
Stage III, IV<br>(*n* = 318)	Referent	1.44 (0.69-3.01)	0.92 (0.42-1.99)	0.61 (0.23-1.57)	0.59 (0.19-1.81	0.13
**BMI (kg/m^2)**						
BMI < 25)<br>(*n* = 160)	Referent	1.98 (0.18-1.52)	1.44 (0.32-4.13)	0.69 (0.13-3.61)	0.77 (0.13-4.85)	0.18
BMI ≥25<br>(*n* = 303)	Referent	1.67 (0.72-3.89)	1.19 (0.46-3.09)	0.63 (0.21-1.90)	0.99 (0.30-3.33)	0.54
**Smoking Status**						
Current/former<br>(*n* = 330)	Referent	0.18 (0.55-2.52)	1.02 (0.47-2.19)	0.51 (0.19-1.35)	0.67 (0.23-1.97)	0.04
Never<br>(*n* = 132)	Referent	0.17 (0.01-2.01)	0.07 (0.00-1.83)	0.04 (0.00-1.14)	0.02 (0.00-0.76)	0.31
**Tumor Site**						
Oropharynx<br>(*n* = 184)	Referent	1.62 (0.43-6.10)	0.66 (0.14-3.18)	5.50 (1.05-28.89)*	1.20 (0.16-9.23)	0.80
Oral Cavity<br>(*n* = 173)	Referent	3.34 (1.00-11.19)	2.24 (0.57-8.73)	0.87 (0.19-4.04)	1.16 (0.23-5.90)	0.54
Larynx<br>(*n* = 94)	Referent	0.72 (0.15-3.53)	0.59 (0.14-2.49)	0.51 (0.06-4.40)	0.66 (0.05-8.37)	0.63

*** *p* <0.0045** [3]

[1] Stratified models adjusted for sex, age, HPV status, tumor stage, tumor site, glycemic load, total fat, mean fruit and vegetable consumption, education status, BMI, smoking, and drinking status.
[2] *p* value for a test of linear trend. Participant dietary fiber intake level was set to the median of the subject's respective quintile. This variable was subsequently modeled as a continuous term using Cox regression.
[3] The level of α = 0.0045 was adjusted using Holm-Bonferonni method for multiple comparisons.

Table S6. Multivariable hazard ratios and their 95% confidence intervals of recurrence risk by pretreatment total whole grains consumption quintiles, stratified by sex, stage, BMI, smoking status, and tumor site.

**Whole grains intake quintile**	1	2	3	4	5	*P*-trend[1]
**Sex**						
Males (n = 344)	Referent	0.72 (0.37-1.39)	0.64 (0.29-1.40)	0.74 (0.35-1.58)	0.59 (0.27-1.30)	0.29
Females (n = 117)	Referent	8.76 (1.21-63.17)	2.26 (0.24-20.89)	18.14 (2.06-159.83)	2.03 (0.26-15.76)	0.83
**Stage**						
Stage 0, I,II (n = 165)	Referent	2.45 (0.50-11.86)	1.67 (0.30-9.31)	3.59 (0.68-19.09)	0.91 (0.12-7.10)	0.31
Stage III, IV (n = 318)	Referent	0.90 (0.46-1.77)	0.75 (0.37-1.53)	1.03 (0.50-2.09)	0.25 (0.25-1.14)	0.14
**BMI (kg/m^2)**						
BMI < 25) (n = 160)	Referent	1.10 (0.38-3.24)	1.04 (0.33-3.25)	1.47 (0.41-5.33)	0.99 (0.28-3.48)	0.99
BMI ≥25 (n = 303)	Referent	0.82 (0.38-1.79)	0.62 (0.27-1.44)	1.07 (0.48-2.37)	0.54 (0.23-1.26)	0.47
**Smoking Status**						
Current/former (n = 330)	Referent	0.78 (0.39-1.54)	0.50 (0.23-1.09)	1.05 (0.53-2.09)	0.54 (0.24-1.19)	0.31
Never (n = 132)	Referent	1.40 (0.15-13.21)	0.17 (0.02-1.69)	0.25 (0.03-2.42)	0.39 (0.05-3.15)	0.48
**Tumor Site**						
Oropharynx (n = 184)	Referent	1.09 (0.30-3.94)	0.43 (0.11-1.66)	1.54 (0.38-6.32)	1.79 (0.43-7.56)	0.48
Oral Cavity (n = 173)	Referent	2.03 (0.66-6.32)	2.85 (0.76-10.67)	1.65 (0.48-5.67)	1.77 (0.52-6.11)	0.54
Larynx (n = 94)	Referent	0.76 (0.18-3.16)	0.28 (0.04-1.77)	0.66 (0.14-3.03)	0.47 (0.08-2.78)	0.44

*** p <0.0045** [3]

[1] Stratified models adjusted for sex, age, HPV status, tumor stage, tumor site, glycemic load, total fat, mean fruit and vegetable consumption, education status, BMI, smoking, and drinking status.
[2] p value for a test of linear trend. Participant whole grains intake level was set to the median of the subject's respective quintile. This variable was subsequently modeled as a continuous term using Cox regression.
[3] The level of α = 0.0045 was adjusted using Holm-Bonferonni method for multiple comparisons.

Table S7. Interaction effects tested in Cox regression models that included all noted covariates from the fully adjusted model.

All-Cause Mortality	
**Fiber**	$P_{interaction}$[1]
Fiber and Sex	0.36
Fiber and Stage	0.11
Fiber and Tumor Site	0.54
Fiber and BMI	0.38
Fiber and Smoking	0.07
**Whole grains**	$P_{interaction}$
Whole grains and Sex	0.19
Whole grains and Stage	0.69
Whole grains and Tumor Site	0.53
Whole grains and BMI	0.29
Whole grains and Smoking	0.71
Recurrence	
**Fiber**	$P_{interaction}$
Fiber and Sex	0.89
Fiber and Stage	0.52
Fiber and Tumor Site	0.57
Fiber and BMI	0.85
Fiber and Smoking	0.14
**Whole grains**	$P_{interaction}$
Whole grains and Sex	0.42
Whole grains and Stage	0.37
Whole grains and Tumor Site	0.08
Whole grains and BMI	0.97
Whole grains and Smoking	0.43
Cancer-Specific Mortality	
**Fiber**	$P_{interaction}$
Fiber and Sex	0.54
Fiber and Stage	0.51
Fiber and Tumor Site	0.57
Fiber and BMI	0.54
Fiber and Smoking	0.32
**Whole grains**	$P_{interaction}$
Whole grains and Sex	0.64
Whole grains and Stage	0.79
Whole grains and Tumor Site	0.14
Whole grains and BMI	0.29
Whole grains and Smoking	0.56

*** *p* <0.0045** [2]

[1] Significance ($p_{interaction}$) of the interaction cross-product term from the nested models was determined using the Likelihood Ratio test.
[2] The level of $\alpha = 0.0045$ was adjusted using Holm-Bonferonni method for multiple comparisons.

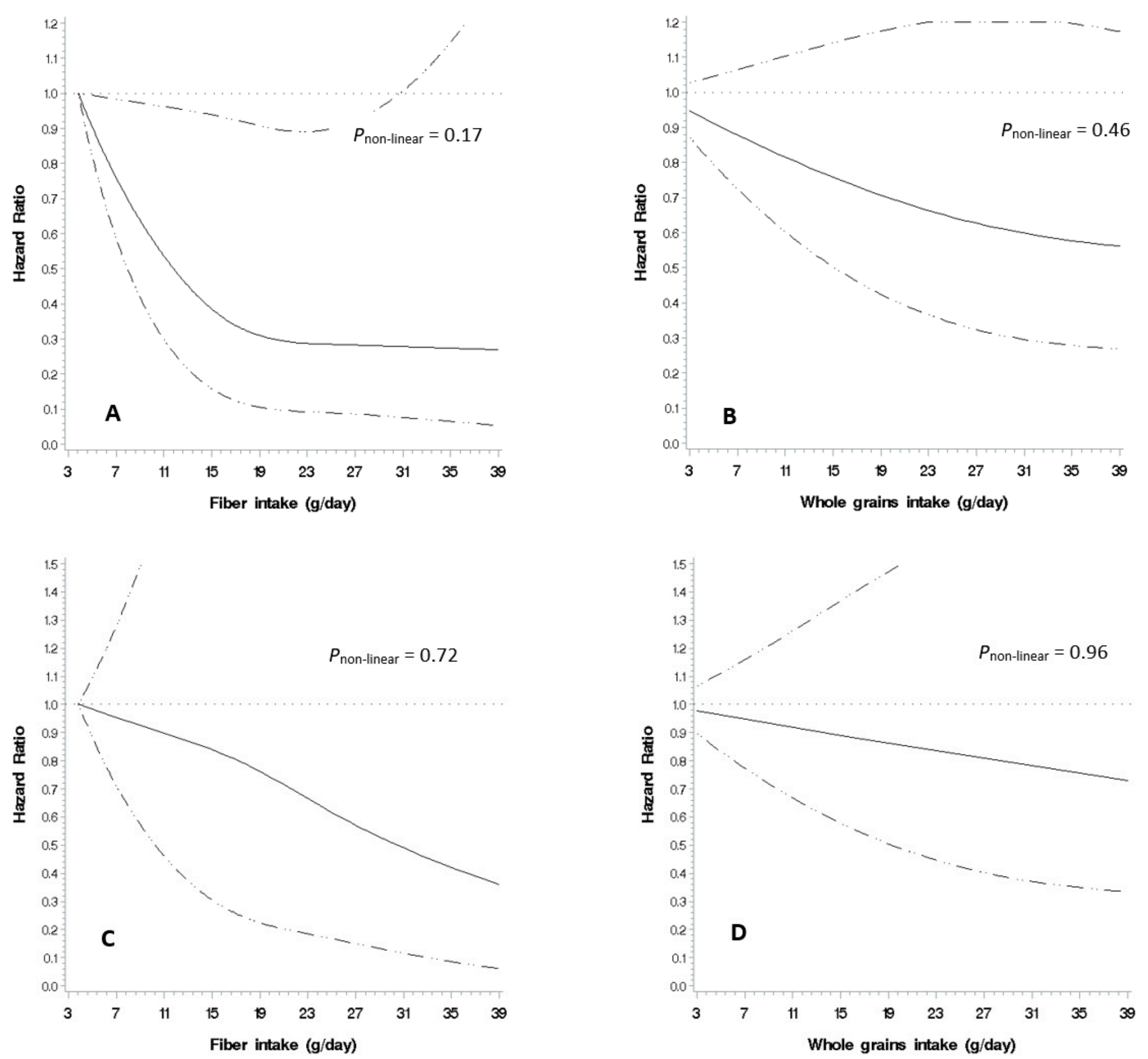

Figure S1. Results from polynomial regression with restricted cubic splines. Curves for the association between dietary fiber (A) or whole grains (B) intake and all-cause mortality are shown as well as recurrence models for dietary fiber (C) and whole grains (D). Hazard ratio estimates were derived using the Cox proportional hazards model, adjusted for sex, age, HPV status, tumor stage, tumor site, education status, mean fruit and vegetable consumption, glycemic load, total fat, BMI, smoking, and drinking status. Solid lines indicate the parameter estimate with dashed lines representing the bounds of the 95% confidence intervals.

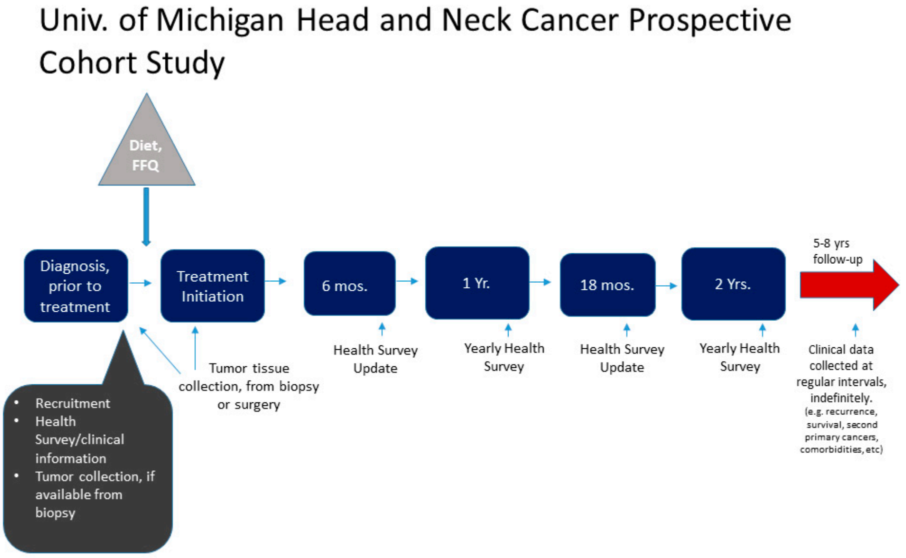

Flow diagram for the Michigan Head and Neck Cancer Prospective Cohort Study
